# Adaptive laboratory evolution of *E. coli* to magnesium chloride stress reveals noncanonical routes to broad chaotropic salt resistance

**DOI:** 10.3389/fmicb.2026.1898632

**Published:** 2026-07-23

**Authors:** Luke A. Fisher, Carleton H. Coffin, Trevor Palmiotto, Catherine A. Royer, Britney E. Schmidt, Jeff S. Bowman, Douglas H. Bartlett

**Affiliations:** 1 Scripps Institution of Oceanography, University of California San Diego, La Jolla, CA, United States; 2 Department of Biological Sciences, Rensselaer Polytechnic Institute, Troy, NY, United States; 3 Department of Earth and Atmospheric Sciences, Cornell University, Ithaca, NY, United States

**Keywords:** adaptive laboratory evolution (ALE), chaotropicity, magnesium chloride, osmotic adaptation, Rcs phosphorelay, reactive oxygen species (ROS), stress response, sugar phosphotransferase system

## Abstract

Extensive research on model microorganisms under kosmotropic sodium chloride stress has established much of what is known about the genetic and molecular mechanisms of osmoregulation. However, genetic adaptation to chaotropic salts such as magnesium chloride remains poorly defined. Chaotropes destabilize biomolecules and can constrain biomanufacturing, underscoring the need to enhance stress tolerance to improve production efficiency. Environmentally, the exceptional chaotropic activity of magnesium chloride brines offers rare opportunities to probe the limits of life, with major implications for bioprospecting and astrobiology. To explore mechanisms of adaptation to high chaotropicity, we used adaptive laboratory evolution (ALE) to isolate *Escherichia coli* strains with improved growth under magnesium chloride stress. Two isolates were obtained which exhibited significantly enhanced growth when exposed to magnesium chloride and other chaotropic salts, but not to sodium chloride. Whole-genome sequencing, label-free quantification, and genetic analysis implicated mutations in the sugar phosphotransferase system, the stringent response, and envelope remodeling as key contributors to magnesium chloride resistance. Mutations in *ptsI*, a sugar phosphotransferase, and *spoT*, encoding the bifunctional (p)ppGpp hydrolase/synthetase, were essential for improved growth under magnesium chloride stress. Interestingly, activation of the regulator of capsular polysaccharide synthesis (Rcs) system, a critical regulator of the osmotic stress response, was required for magnesium chloride tolerance in the ancestral strain but became dispensable in the evolved strains. Our report identifies multiple strategies used by *E. coli* to overcome magnesium chloride stress.

## Introduction

To survive and propagate, prokaryotes must be able to adapt to rapidly fluctuating physical and chemical conditions in their environment such as temperature, pH, and redox state. In *E. coli*, cells respond to hyperosmotic shock by enhancing potassium transport and importing or synthesizing compatible solutes to become iso-osmotic with their environment and maintain turgor pressure (Arense et al., 2010; Csonka, 1989; Gunasekera et al., 2008; Le Rudulier et al., 1984; Wood, 1999;). Osmoregulation in *E. coli* is largely mediated by membrane-affiliated signal transduction pathways and transporters such as Kdp, EnvZ/OmpR, Trk, ProP, and ProU (Sleator and Hill, 2002). Osmotic stress and other cell surface and peptidoglycan altering conditions also activate the Rcs phosphorelay system, which helps prevent desiccation and mitigates membrane stress by the production of colanic acid capsular polysaccharide (Majdalani and Gottesman, 2005; Petchiappan et al., 2024; Sledjeski and Gottesman, 1996; Wall et al., 2018).

By contrast, far less is known about how prolonged osmotic stress drives genetic adaptation for sustained growth rather than acute survival. Efforts to engineer salt-tolerant *E. coli* strains have been made (Cesar et al., 2020; Dragosits et al., 2013; Purvis et al., 2005; Rozwadowski et al., 1991; Winkler et al., 2014; Wu et al., 2014) but have provided only a glimpse into the genetic basis of this multifaceted phenotype, underscoring the need for detailed molecular analyses. Moreover, most studies investigating osmotic stress resistance/adaptation in prokaryotes have focused on sodium chloride or other kosmotropes such as magnesium sulfate (Nepal and Kumar, 2020), whereas adaptation to chaotropic salts such as magnesium chloride–which imposes fundamentally different stressors on biomolecules–remains largely unexplored (Hallsworth et al., 2003; Heinz et al., 2019). Chaotropic agents, or chaotropes, are typically large ions with low charge density that disrupt the hydrogen-bonding network of water, promoting protein unfolding, enzyme inhibition, and membrane solubilization (Ball and Hallsworth, 2015; Collins, 1997; Cray et al., 2013; Hallsworth et al., 2007; Timson, 2020). Chaotropicity is a central determinant of product tolerance in biofuel-producing microorganisms, as numerous biofuel products act as chaotropes that can limit production efficiency (Cray et al., 2015). Beyond industrial relevance, chaotropic environments inform the limits of life on Earth and, potentially, elsewhere. Naturally occurring magnesium chloride brines are rare and include highly stratified deep-sea hypersaline anoxic basins (DHABs), where their exceptional chaotropicity is largely considered incompatible with life (Hallsworth et al., 2007; Klempay et al., 2021). Thus, elucidating the microbial strategies of coping with chaotropic stress is critical for both industrial strain engineering and stress biology fundamentals.

In this work, adaptive laboratory evolution (ALE) was used to select for *Escherichia coli* strains with improved growth at elevated MgCl_2_ concentrations. Two ALE-derived strains with enhanced magnesium chloride tolerance were obtained and characterized. The results connect chaotrope adaptation to changes in cell envelope structure and central metabolism, with potential implications for ROS-associated stress physiology described in previous models of bacterial cell death (Kohanski et al., 2007; Zeng et al., 2022).

## Materials and methods

### Growth conditions and adaptive laboratory evolution

*E. coli* strains were routinely grown in Luria-Broth (LB) shaking at 37 °C unless indicated otherwise. For solid format plates, the LB was amended with 17 g L^−1^ agar. When required, the medium was amended with 10 μg/mL gentamicin, 100 μg/mL ampicillin, 25 μg/mL chloramphenicol, or 50 μg/mL kanamycin from filter sterilized stock solutions. Adaptive laboratory evolution was initiated using three replicate lineages (A, B, and C) of *E. coli* substr. MG1655 grown in LB medium amended with 150 mM MgCl_2_, added as magnesium chloride hexahydrate from a sterile 4 M stock solution. The parental MG1655 strain (Blattner et al., 1997) served as the non-evolved salt sensitive ancestor, as it grows robustly in LB but exhibits impaired growth in 150 mM MgCl_2_ LB. Cultures that reached an optical density evaluated at 600 nm (OD600) of at least 0.35 were diluted 1:100 into fresh MgCl_2_ amended LB for the next transfer. OD600 of each lineage was measured using a Spectronic 20+ spectrophotometer (Thermo Fisher Scientific, Waltham, MA, United States). Transfers were initially performed daily during the early stages of ALE but required up to ~48 h of incubation to reach the target OD600 as magnesium chloride concentrations increased. With each transfer, MgCl_2_ concentrations increased by 10–25 mM MgCl_2_ until a final concentration of 350 mM was reached. Each subculture was assigned a new lineage number (A1, B1, and C1) at every transfer and was cryopreserved at −80 °C in 15% w/v sterile glycerol. Transfers of the A lineage were eventually discontinued due to its consistently poorer growth in MgCl_2_ amended LB compared to the B and C lineages. After approximately 230 generations (estimated from OD600 between transfers using the 1:100 dilution factor and culture volume) a clonal isolate from the B and C lineages were obtained by streaking onto magnesium chloride amended agar plates. These isolates were used for downstream analyses and are hereafter referred to as strains LFB34 and LFC35. See Table 1 for the full list of strains and plasmids used in this study.

**Table 1:** Strains and plasmids used in this study.

Strain	Description	Source
MG1655	Wild-type ancestral *E. coli* K-12 strain	Coli Genetics Stock Center (CGSC)
DH5α	*recA*^−^ cloning strain	CGSC
BW25113	Keio collection parental strain	CGSC
JW1946	BW25113, *drpB*::kan	CGSC
JW2409	BW25113, *ptsI*::kan	CGSC
JW2410	BW25113, *crr*::kan	CGSC
JW0627	BW25113, *dacA*::kan	CGSC
LF01	MG1655, Δ*rcsB*	This study
LF02	MG1655, Δ*rcsC*	This study
LF03	MG1655, Δ*rcsD*	This study
LF04	MG1655, Δ*rcsF*	This study
LF05	MG1655, *rcsB* (G61S)	This study
LF08	MG1655, *spoT*::kan	This study
LF09	MG1655, *ptsI*::kan	This study
LFB34	MgCl_2_ evolved MG1655	This study
LFC35	MgCl_2_ evolved MG1655	This study
LFB341	LFB34, *rcsBCD*::kan	This study
LFC351	LFC35, *rcsBCD*::kan	This study
LFB342	LFB34, wild-type *rcsD*	This study
Plasmids and phage		
pEE3	Complementation vector, Kan^R^	Lauro et al. (2005)
pEE3-spoT	pEE3 with wild-type MG1655 *spoT* gene	This study
pEE3-ptsI	pEE3 with wild-type MG1655 *ptsI* gene	This study
pKD46	Temperature sensitive arabinose inducible helper plasmid encoding λRed genes (γ, β, and exo), Amp^R^	Datsenko and Wanner (2000)
pKD4	Source of kanamycin cassette	Datsenko and Wanner (2000)
pCP20	Temperature sensitive helper plasmid with thermal induction of FLP synthesis, Amp^R^	Cherepanov and Wackernagel, (1995)
pSIM6	Homologous recombination vector, Amp^R^	Chan et al. (2007)
pACBSR	Arabinose inducible helper plasmid controlling I-*Sce*I and λRed functions, Cm^R^	Herring et al. (2003)
pRed_Cas9_recA	Temperature sensitive arabinose inducible Cas9 λRed functions, Kan^R^	Zhao et al. (2016)
pRedCas9-rcsBG61S	pRedCas9, gRNA-*rcsB*, donor *rcsB* G61S	This study
P1*vir*	Transducing phage	CGSC

### Growth assessments

Overnight cultures grown in LB were diluted 1:100 into sterile media and grown in triplicate in 48-well plates incubated at 37 °C. Growth was monitored using a SpectraMax^®^ iD3 plate reader which measured OD600 continuously (Molecular Devices, San Jose, CA, United States). To assess growth in minimal medium, cells from overnight LB cultures were washed three times *via* centrifugation (9,000 rcf for 1-min) with M9 minimal salts broth (Teknova, Hollister, CA, United States). Washed cells were diluted 1:100 into triplicate glass culture tubes containing M9 amended with 0.4% w/v glycerol, pyruvate, glucose, maltose, fructose, lactose, or casamino acids which were added from filter sterilized stock solutions.

### Cell size analysis

Cell size analysis was performed using fluorescence microscopy to generate images of each strain using the signal from the homogeneously distributed intrinsic fluorophore, NADH. To obtain accurate cell size information, cells were imaged in mid logarithmic phase (under balanced growth conditions). Overnight cultures grown in LB or MgCl_2_ amended LB near their respective upper magnesium chloride growth limit (250 mM for MG1655, and 350 mM for LFB34 and LFC35) were diluted 1:100 into fresh media of the same osmotic strength and subsequently grown to mid log-phase (OD600 ~0.4–0.5). Cells were then prepared for imaging as described previously (Coffin et al., 2024) with minor modifications. For cultures grown in MgCl_2_, the M9 was supplemented with the same MgCl_2_ concentration during transfers and when preparing the 2% agarose pads to prevent osmotic shock. The pad was mounted in an autofluor for imaging on an ISS Alba fast scanning mirror fluctuation microscope furnished with a Mai Tai^®^ TI:Sapphire 2-photon laser. 750 nm excitation light (power ~38 mW) was focused through a 60 × 1.2 NA water immersion objective onto a No. 1 coverslip with the agarose pad to excite NADH *via* 2-photon excitation and generate a fluorescence signal with distinct cell boundaries that could be individually segmented. All images were captured in 20 μm x 20 μm fields of view (FOVs) and analyzed using a custom Matlab script which uses an AI model trained using *E. coli* MG1655 data to quickly and accurately mask cells after image denoising using block-matching and 3D filtering (BM3D). After cell segmentation, the volume of each cell was calculated. For details, see the Supplementary material.

### Measurement of ROS

Reactive oxygen species (ROS) were measured using the ROS-sensitive fluorescent dye Carboxy-H2DCFDA (Thermo Fisher Scientific, Waltham, MA, United States), following previously described methods (Bass et al., 1983; Brandt and Keston, 1965; Hong et al., 2019; Zeng et al., 2022), with fluorescence measured using a SpectraMax^®^ iD3 plate reader (Molecular Devices, San Jose, CA, United States). Overnight cultures of MG1655, LFB34, and LFC35 were diluted 1:100 into 5 mL LB cultures and grown until early exponential phase (OD600 ~0.25) at 37 °C. Cells were then washed twice with 1X PBS by centrifugation at 9,000 x g for 2-min. Carboxy-H2DCFDA was then added to a final concentration of 10 μM, and samples were incubated in the dark at 22 °C for 10 min. H_2_O_2_ was then added to a final concentration of 2 mM and fluorescence intensity (492 Ex./535 Em.) was measured over time. No-dye controls and dye-treated cultures without H_2_O_2_ were included as controls, and both showed little fluorescence.

### Whole genome sequencing and identification of mutations

Genomic DNA from the ancestor (MG1655), LFB34, and LFC35 was isolated using the DNeasy^®^ UltraClean^®^ Microbial Kit following the manufacturer's instructions (QIAGEN, Hilden, Germany). DNA was sent to Novogene (Sacramento, CA, United States) for Illumina library construction and sequencing on a NovaSeq 6,000 PE150 (Illumina, San Diego, CA, United States). Read quality was assessed using FastQC (Andrews, 2010) followed by read trimming using PRINSEQ v0.20.4 (Schmieder and Edwards, 2011). Mutations were then identified from trimmed FASTQ files using *breseq* (Deatherage and Barrick, 2014) using the *E. coli* str. K-12 substr. MG1655 genome as the reference file (Blattner et al., 1997). Mutations of interest were verified by polymerase chain reaction (PCR) with gene specific primers and Sanger sequencing (Retrogen, San Diego, CA, United States).

### Label-free quantification

Overnight cultures from colony isolates grown in 5 mL of LB or 250 mM MgCl_2_ LB (herby referred to as LB250) were diluted 1:200 into 5 mL of fresh medium and allowed to grow to an OD600 of 0.2 to ensure steady state growth. Steady state cultures were diluted 1:200 in triplicate into 5 mL of medium and grown to mid log-phase (MG1655: ~0.4 for LB and ~0.2 for LB250; LFB34 and LFC35: ~0.4 for LB and LB250). Cells were then harvested *via* centrifugation at 10,000 rcf for 3 min at 4 °C and subsequently washed twice with 10 mL of ice-cold 1X PBS buffer. Cell pellets were snap frozen in liquid nitrogen and stored at −80 °C. Samples were then sent to the UC San Diego Biomolecular and Proteomics Mass Spectrometry Facility (BPMSF) for protein extraction, mass spectrometry, and statistical analysis. Methods for protein extraction, mass spectrometry, and acquisition of the DIA library can be found in the Supplementary material.

### Analysis of proteomics data

Peptide identifications were performed in PEAKS Studio 11 version 12.5 (Bioinformatics Solutions, Ontario, Canada) against the *Escherichia coli* K-12 substr. MG1655 proteome (GenBank accession no. U00096.3). Peptides passing a 1% false discovery rate (FDR) cutoff were used to generate the data independent acquisition (DIA) library. Protein abundance comparisons were then performed in PEAKS Studio 11 using TIC-normalized LFQ peak area values from three biological replicates per strain (MG1655, LFB34, and LFC35) per condition (LB and LB250). Differentially abundant proteins were identified using the PEAKS built-in statistical analysis tool, with differential abundance assessed by ANOVA and corrected for multiple testing using the FDR approach implemented in PEAKS. Proteins with a ±1 log2 fold change (2 fold upregulated or 0.5 downregulated) and an ANOVA based significance value (-10 log *P*-value) ≥ 15, corresponding to *p* ≤ 0.0316, were used as thresholds for differentially expressed proteins (DEPs). Additional details on the PEAKS algorithms are available in Zhang et al. (2012). Proteins were assigned further identifiers using the Uniprot ID mapping tool and EcoCyc (Keseler et al., 2011). Clusters of Orthologous Genes (COG) identifiers were assigned using IMG (Chen et al., 2021). Gene lists were downloaded from Regulon DB or EcoCyc to classify proteins by regulator or GO term, respectively (Keseler et al., 2011; Salgado et al., 2006). Functional annotation clustering of DEPs was performed using the online tool DAVID (The Database for Annotation, Visualization, and Integrated Discovery) with default parameters and medium stringency (Dennis et al., 2003). DAVID annotation clusters with enrichment scores ≥1 were analyzed in further detail if they contained at least one enriched term with a Benjamini-adjusted *p* < 0.05. In parallel, DEPs were also analyzed using the STRING database (Szklarczyk et al., 2023) with default settings. The results of these analyses can be found in Supplementary Files 1, 2.

### Genetic manipulations

Chromosomal gene deletions were made using lambda Red recombineering by replacing genes of interest with kanamycin cassettes amplified *via* PCR using pKD4 plasmid DNA as a template (Datsenko and Wanner, 2000). Gene knockouts were identified using colony PCR and gel electrophoresis. To assess the impact of *breseq* identified mutations on MgCl_2_ growth tolerance, individual mutations were introduced *via* complementation, CRISPR/Cas9 mutagenesis, or seamless insertion. For complementation experiments, genes of interest and their promoters were PCR amplified and TA cloned into the low copy complementation vector pEE3 (Lauro et al., 2005). pEE3 is a kanamycin resistant TA-cloning vector with a multiple cloning site located within *lacZ*, enabling easy insert screening. The empty pEE3 vector was transformed into the corresponding strain background and used as the vector control for each complementation experiment. CRISPR/Cas9 mutagenesis was conducted following the methods of Zhao et al. (2016) and scarless knock-in insertion mutations were introduced based on methods described in Lee et al. (2009) and Diner et al. (2011). Genetic manipulation of LFC35 was limited by extremely poor recovery of plasmid-complemented strains and complete resistance to P1 phage. Therefore, targeted genetic analyses in LFC35 were limited, and were focused on LFB34 and MG1655-derived backgrounds. All primers used in this study were ordered from Integrated DNA Technologies (IDT™, San Diego, CA, United States), and their sequences are listed in Supplementary Table S1.

## Results

### ALE derived MgCl_2_ tolerant *E. coli* strains exhibit broad resistance to chaotropic salts and distinct morphologies

**Figure 1:**
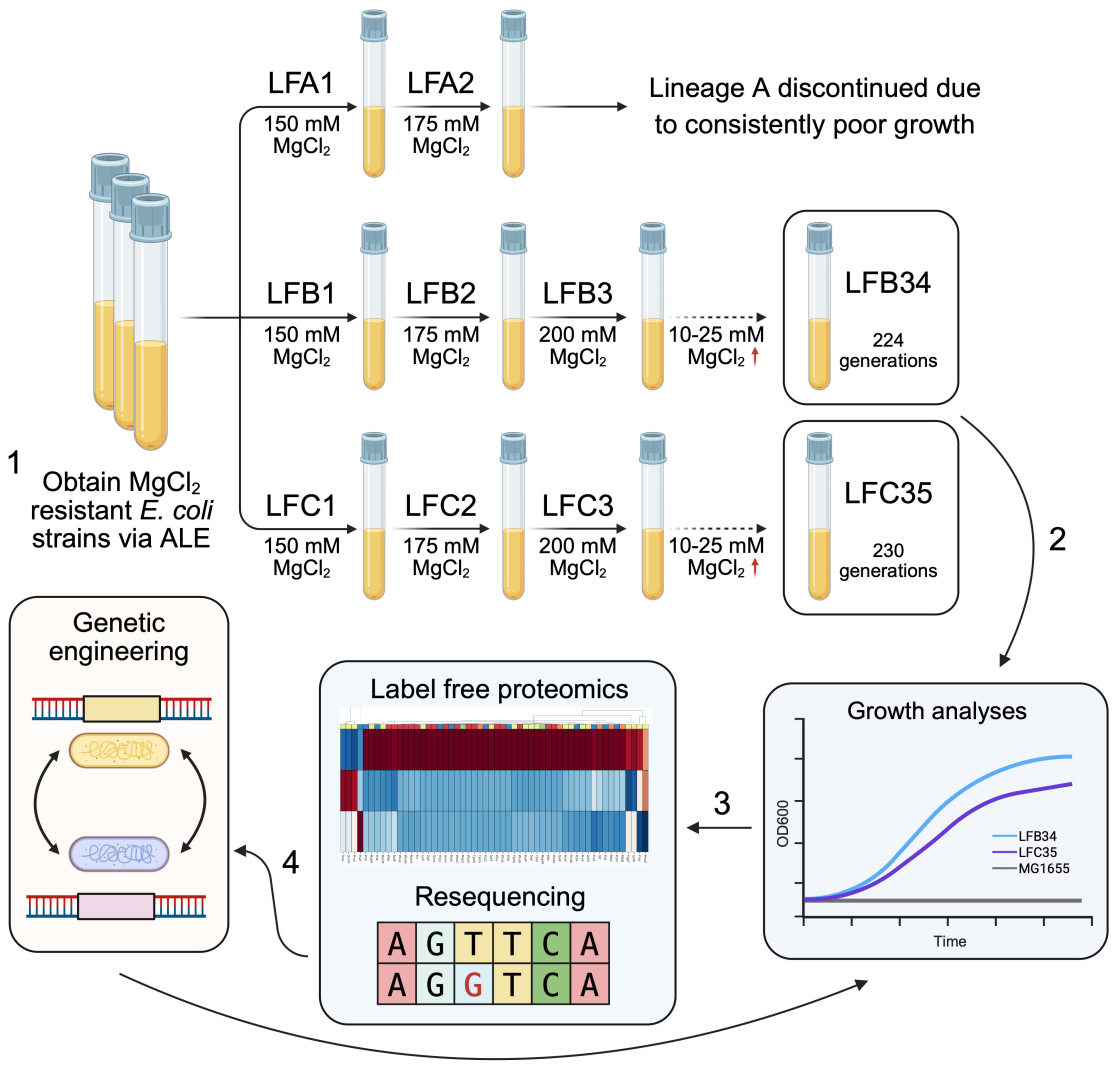
Experimental ALE approach and downstream analyses. (1) Triplicate *E. coli* lineages were serially passaged in LB medium with increasing concentrations of MgCl_2_. Cultures were initially grown in LB amended with 150 mM MgCl_2_, and the MgCl_2_ concentration was increased by 10–25 mM at each transfer. Lineage A was discontinued due to consistently poor growth. (2) Growth of the evolved strains was assessed in the presence of a variety of osmotic stressors. (3) The response to elevated MgCl_2_ in the ancestral and evolved strains was characterized using label-free quantification (heat map) and whole-genome resequencing was conducted using *breseq* (sequence alignment cartoon) to identify mutations that arose during ALE. (4) Select mutations were investigated using Recombineering and/or CRISPR/Cas9 mutagenesis, followed by evaluation of growth under MgCl_2_ stress. Created with BioRender.com.

**Figure 2:**
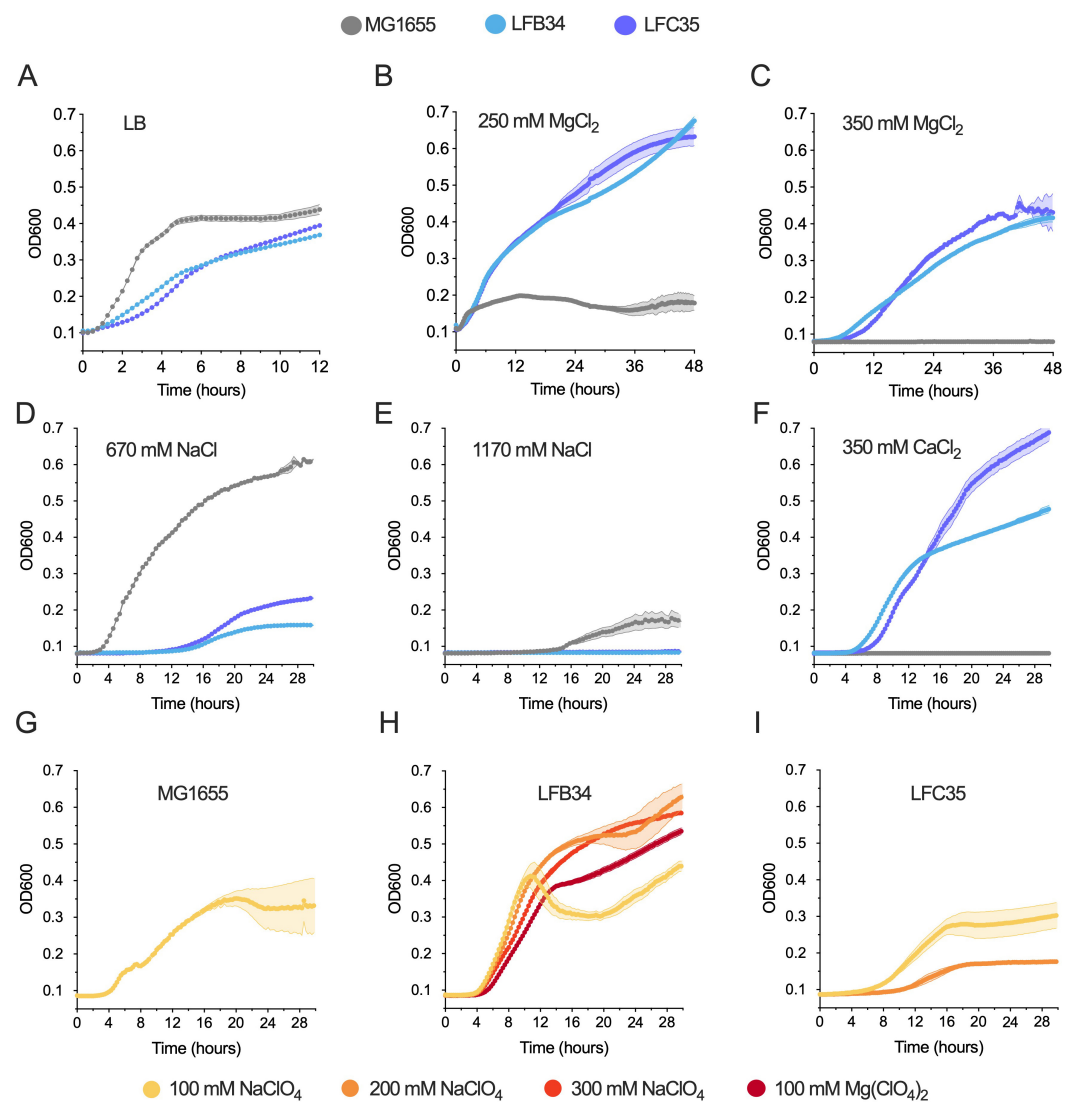
Growth curves of the ancestral and evolved strains under various salt stressors. MG1655 (gray), LFB34 (blue), and LFC35 (purple) were grown in LB **(A)**, 250 mM MgCl_2_ LB **(B)**, 350 mM MgCl_2_ LB **(C)**, 670 mM NaCl LB **(D)**, 1170 mM NaCl LB **(E)**, and 350 mM CaCl_2_ LB **(F)**. Growth curves of MG1655 **(G)**, LFB34 **(H)**, and LFC35 **(I)** grown in 100, 200, and 300 mM sodium perchlorate LB, and 100 mM magnesium perchlorate LB. Points represent the mean of three biological replicates and shaded areas show one standard deviation.

**Figure 3:**
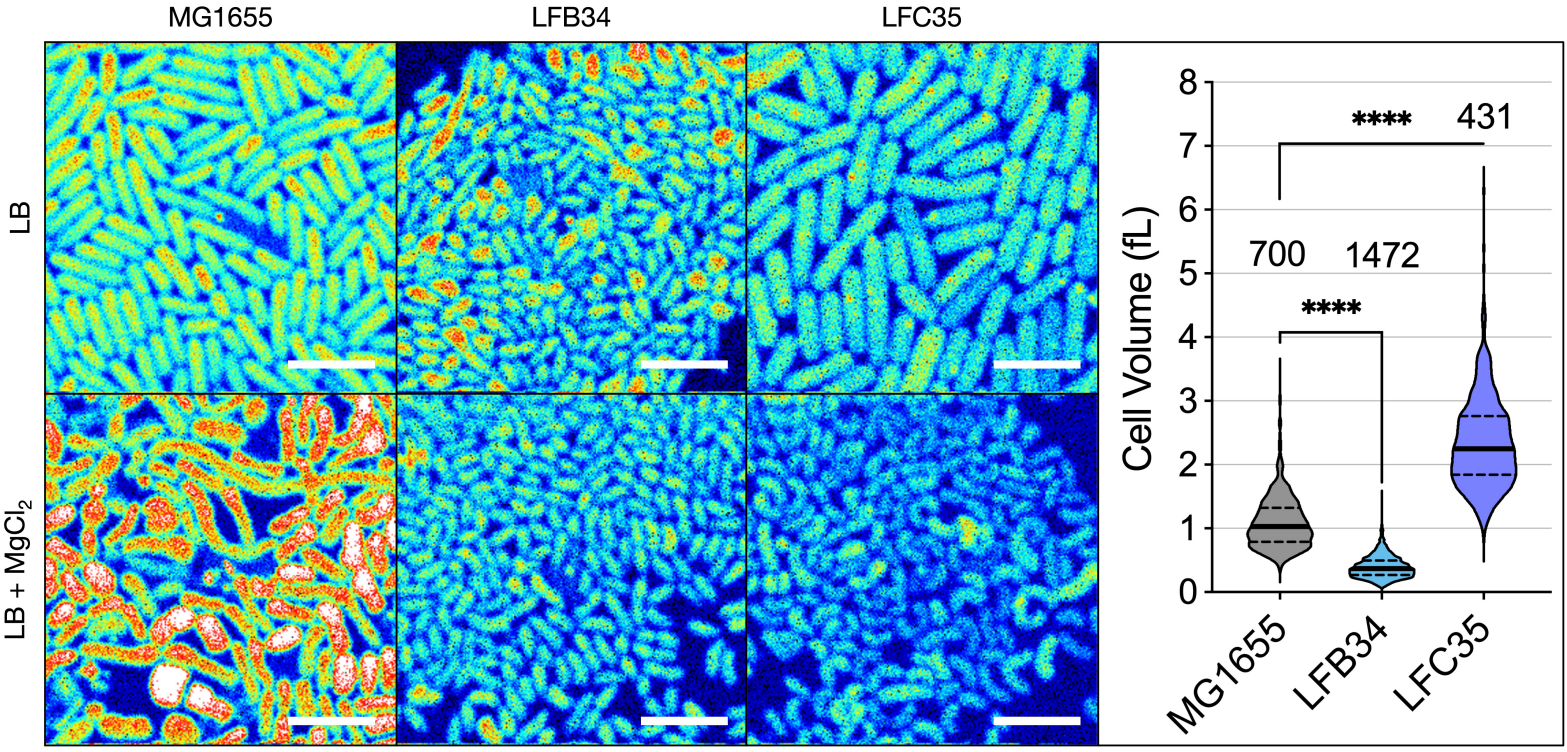
Cell morphology and volume of the ancestral and evolved strains. On the left, representative fluorescence images of strains, indicated by columns, grown to exponential phase in the absence (LB, top) or presence (LB + 250 mM or 350 mM MgCl_2_, bottom) of magnesium chloride. Scale bars are 5 μm. Violin plots on the right show the volume distributions (y-axis) of individual cells captured from eight FOVs in the LB condition. Values on the plot indicate the number of counted cells, the solid line shows the mean, and the dashed lines represent the upper and lower quartiles. Cell volumes were compared by ordinary one-way ANOVA followed by Dunnett's multiple comparisons test against MG1655. *n* = 700, 1,472, and 432 cells for MG1655, LFB34, and LFC35, respectively (****, *P* ≤ 0.0001). Because the analysis software requires straight, rod-shaped cells to accurately calculate cell volume, MG1655 and LFC35 could not be analyzed after growth in LB supplemented with MgCl_2_.

Three independent *E. coli* MG1655 lineages were serially passaged in LB with progressively increasing magnesium chloride concentrations (Figure 1). Lineage A was discontinued due to consistently poor growth, but after approximately 230 generations of ALE, clonal isolates were obtained and designated strains LFB34 and LFC35 (Luke Fisher isolator, lineages B and C, transfers 34 and 35, respectively). LFB34 and LFC35 were characterized by reduced growth rates in LB and notable growth defects in minimal M9 medium (Figure 2A; Supplementary Figure S1). The evolved strains showed significantly enhanced growth in 250 mM MgCl_2_ LB (LB250) and were able to grow at concentrations up to 350 mM (LB350; Figures 2B, C). Intriguingly, LFB34 and LFC35 exhibited significantly impaired growth relative to MG1655 in sodium chloride amended LB (Figures 2D, E). Both strains displayed improved growth in LB amended with other chaotropic salts, including calcium chloride, sodium perchlorate, and magnesium perchlorate, suggesting specific adaptation to chaotropic salts (Figures 2F-I). Live cell imaging in LB and LB250 (for MG1655) or LB350 (for LFB34 and LFC35) revealed significant differences in size and morphology between strains and conditions (Figure 3). In the absence of MgCl_2_, LFB34 cells were smaller than MG1655 (mean cell volumes: 0.397 fL vs. 1.01 fL, respectively), whereas LFC35 cells were enlarged (2.35 fL). However, when grown in the presence of magnesium chloride, MG1655 and LFC35 underwent dramatic cell size and morphology changes. MG1655 displayed a classical filamentation response to stress. For LFC35, cell length and width decreased, and for most of the cells, the morphology shifted from rod shaped to “C” shape. Because the image analysis software requires straight rod shaped cells to calculate cell volume, quantitative volume distributions could not be obtained for MG1655 or LFC35 cells grown in MgCl_2_ amended LB. Nevertheless, the representative images indicate distinct morphology changes in the ancestor and evolved strains, suggesting that adaptation to MgCl_2_ involves altered cell size control.

### The proteomic profiles of the evolved strains vary considerably compared to MG1655

**Figure 4:**
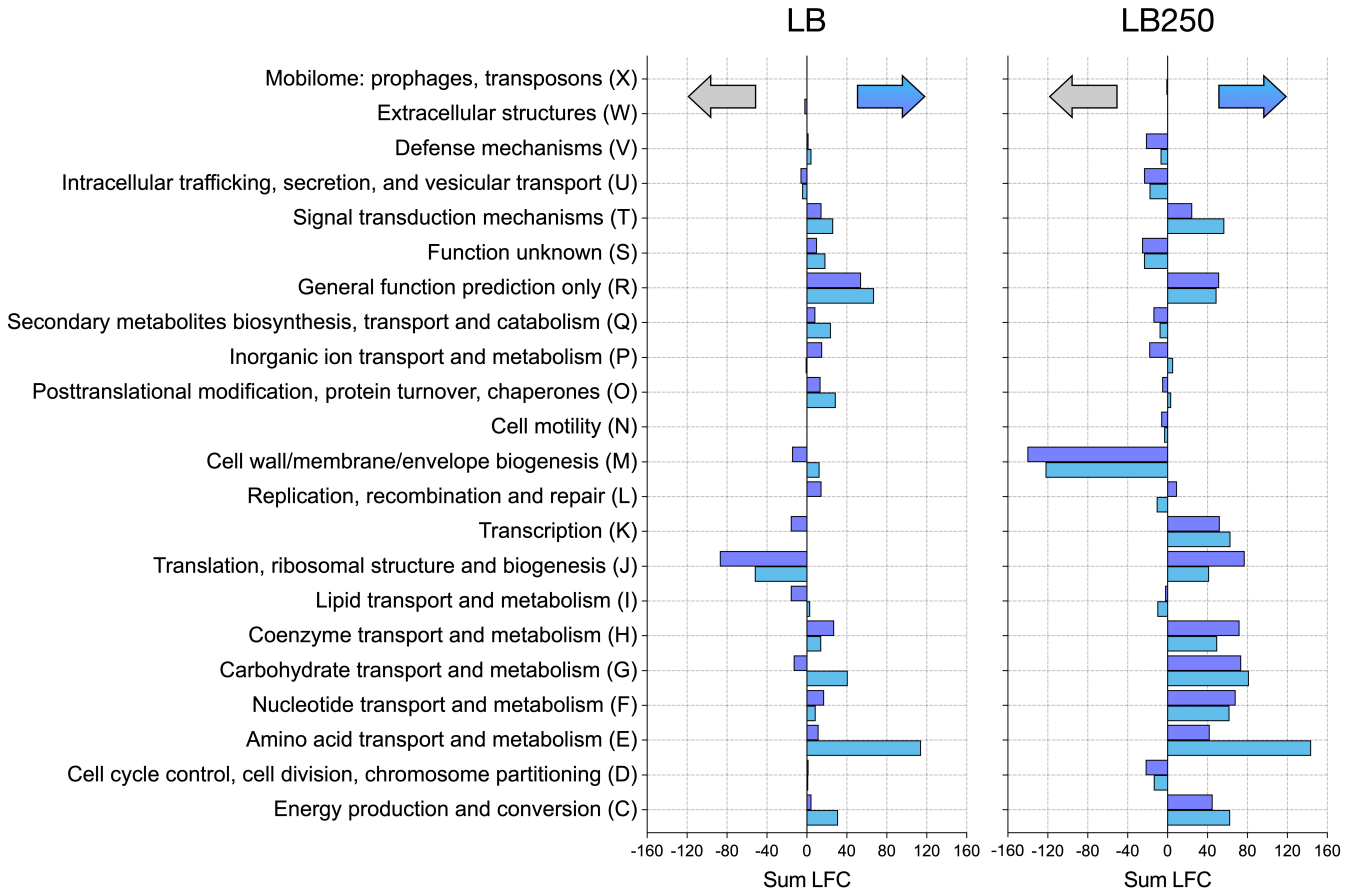
Sum log2 fold change (Sum LFC) of DEPs classified by COG function. Positive values show upregulation in the LFB34 (blue bars) and LFC35 (purple bars) compared to MG1655 represented by the colored arrow in the LB condition **(left)** and the LB250 condition **(right)**. Negative values indicate upregulation in MG1655, represented by the gray arrow, compared to the evolved strains. This visualization was used to identify broad directional trends among functional groups and should not be interpreted as a normalized enrichment statistic, because summed values can be influenced by COG category size and by cancellation among proteins with opposing fold change directions. See Supplementary File 3 for the full list of DEPs.

To gain a more detailed picture of the physiological state of each strain, we compared the proteomic profiles of MG1655, LFB34, and LFC35 grown in LB with and without MgCl_2_. Under LB250 conditions, 605 and 615 proteins were upregulated in LFB34 and LFC35, respectively, relative to MG1655. Among these upregulated proteins, 465 were shared between the evolved strains. STRING analysis of these 465 proteins revealed significant enrichment of proteins in molecular function GO categories GO:0043169 (cation binding), GO:0046872 (metal ion binding), and GO:0046913 (transition metal ion binding), primarily associated in the cytosol. Metal ion binding pathways have previously been implicated in cellular responses to osmotic and oxidative stress, and their upregulation found here could suggest a stress adaptation strategy (Barrientos-Moreno et al., 2020; Winkler et al., 2014; Zorraquino et al., 2016). To broadly summarize the direction of proteomic changes across functional categories, DEPs were assigned to COG categories and visualized by summing log2 fold change values within each category (Figure 4). This analysis was used as a descriptive overview of net category level trends and was not interpreted as a normalized enrichment statistic, as summed log2 fold change values can be influenced by the number of DEPs assigned to each category. With this limitation in mind, the most prominent trend was the strong net increase in COG category M proteins in MG1655 upon growth in LB250, indicating an elevated abundance of proteins associated with colanic acid production, exopolysaccharide biosynthesis, cell wall, membrane, and envelope biogenesis during MgCl_2_ stress (Supplementary Figure S2; Supplementary Table S2). In LB, COG categorization of DEPs showed increased abundance of translation and ribosome associated proteins in MG1655 (Figure 4), consistent with functional annotation clustering analysis indicating enrichment of ribosome and ATP synthase subunits (Supplementary Table S2; Supplementary File 1). For the full list of DEPs and comparisons, see Supplementary File 3.

**Figure 5:**
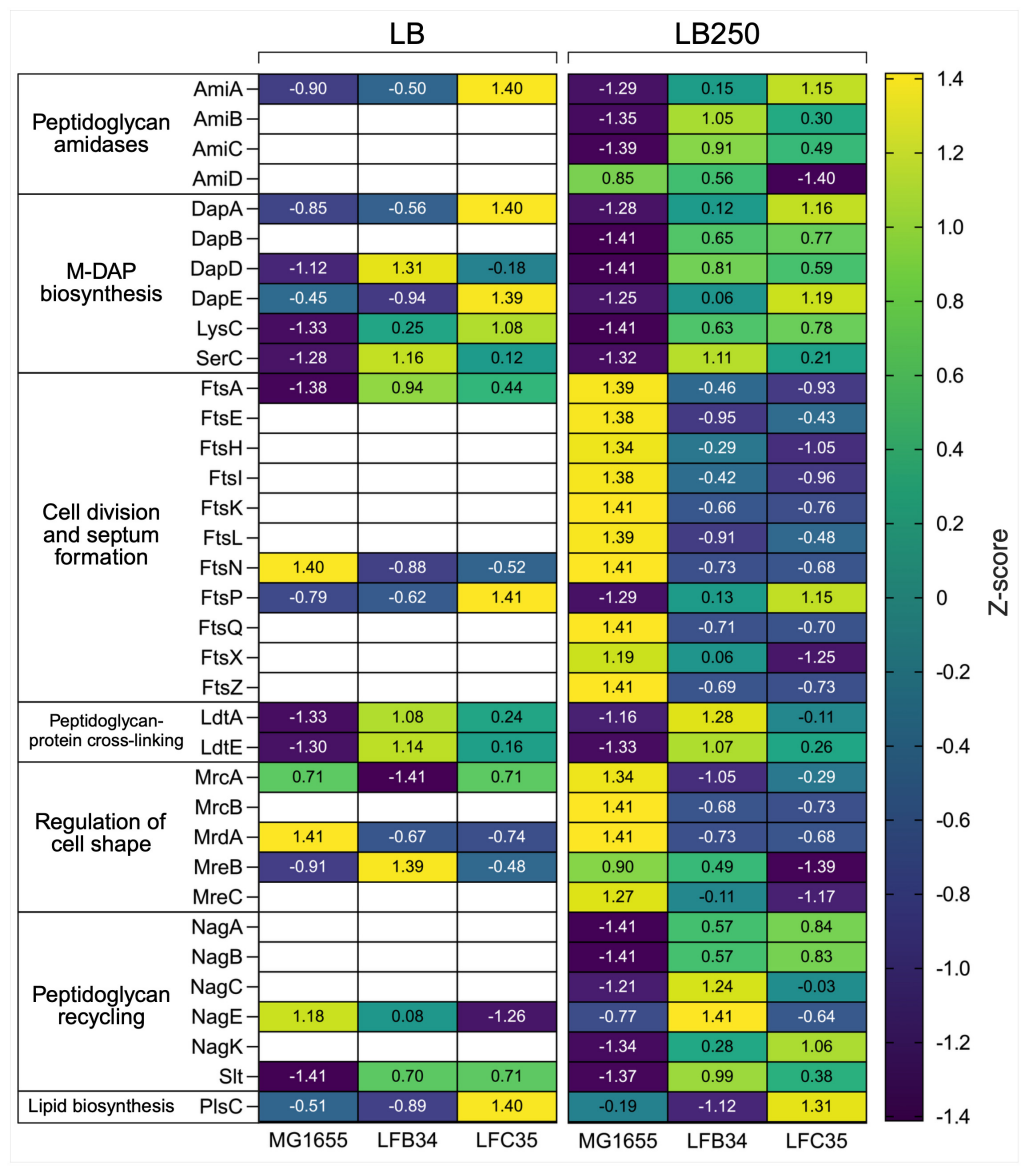
Select cell envelope associated proteomic changes under growth in LB conditions **(left)** and LB250 conditions **(right)**. Heat maps display row-wise Z-scores of log2 transformed protein abundance values for MG1655, LFB34, and LFC35 (columns). Proteins were organized by functional categories related to phospholipid biosynthesis, cell division, and peptidoglycan biosynthesis, maturation, and recycling. Yellow and purple indicate relatively higher and lower abundance, respectively, for each protein across strains. In the LB comparison, empty cells indicate proteins that were either not detected or did not meet the differential abundance cutoff, emphasizing significant abundance changes in cell envelope associated proteins upon growth in MgCl_2_ amended LB. See Supplementary File 3 for the full list of DEPs.

The altered cell morphology of the evolved strains, together with the enrichment of proteins implicated in envelope maintenance, prompted further examination of proteins associated with peptidoglycan biogenesis and cell morphogenesis. Levels of proteins required for synthesizing the peptidoglycan precursor meso-diaminopimelate (DapA, DapB, DapD, DapE, LysC, and SerC) were significantly enriched in both evolved strains compared to MG1655 (Figure 5; Supplementary Table S2). Several proteins involved in peptidoglycan remodeling and recycling were also elevated in the evolved strains, including LdtA/LdtE, AmiA–C, Slt, and several Nag (N-acetylglucosamine; NAG) proteins (Figure 5; Supplementary Table S2; Dijkstra and Thunnissen, 1994; Heidrich et al., 2001; Magnet et al., 2007). NAG is a critical component of peptidoglycan, and mutations in the *nag* genes have been implicated in membrane stress adaptation (Atsumi et al., 2010; Park and Uehara, 2008; Winkler et al., 2014). Thus, it is feasible that the increased abundance of Nag proteins in the evolved strains could impact cell wall structure and contribute to osmotic stress tolerance. Levels of Fts proteins were significantly higher in MG1655 which may reflect compromised divisome function under envelope stress, consistent with the pronounced filamentation observed during magnesium chloride exposure. In contrast, FtsP, which is required for cell division under stress (Samaluru et al., 2007), was significantly upregulated in the evolved strains, suggesting that LFB34 and LFC35 may better maintain division under elevated magnesium chloride conditions.

### The Rcs system is required for MgCl_2_ tolerance in MG1655 but dispensable in the evolved strains

To identify genetic changes underlying the evolved MgCl_2_ tolerant phenotypes, the genomes of MG1655, LFB34, and LFC35 were re-sequenced. In addition to seven mutations shared among the ancestral and evolved strains, LFB34 and LFC35 contained thirteen and fourteen mutations, respectively. Detailed descriptions and annotations can be found in Table 2. These mutations implicated genes associated with menaquinone biosynthesis, envelope structure, peptidoglycan remodeling, phospholipid biosynthesis, carbohydrate metabolism, the stringent response, vitamin biosynthesis, and Rcs signal transduction. Mutations in genes associated with the Rcs system were of particular interest because of its established role in envelope and osmotic stress responses (Majdalani and Gottesman, 2005; Sledjeski and Gottesman, 1996; Takeda et al., 2001; Wall et al., 2018). The Rcs phosphorelay is activated by osmotic stress and regulates capsular polysaccharide synthesis and late-stage biofilm development, with RcsB, RcsC, RcsD, and RcsF required for pathway function. In the LFB34 genome, a loss-of-function MOB (mobile element) insertion was identified in the coding region of *rcsD*, which encodes the RcsD phosphotransferase (Takeda et al., 2001). In the LFC35 genome, a single nucleotide polymorphism (SNP) was identified in the coding region of *rcsB*, which encodes the DNA-binding transcriptional activator RcsB (Chen et al., 2001), resulting in a non-conservative glycine to serine substitution (G61S).

**Table 2:** Nature and descriptions of genetic mutations found in the genomes of the ancestral and evolved strains identified using *breseq* (Deatherage and Barrick, 2014).

Evidence	Position	Mutation	Annotation	Gene	Description
Mutations identified in MG1655, LFB34, and LFC35					
MC JC	257,908	Δ776 bp		*insB9*–*[crl]*	*insB9, insA9, [crl]*
RA	640,491	G → A	G247S (GGT → AGT)	*ahpF*	Alkyl hydroperoxide reductase, AhpF component
JC	1,399,468	(TGATGCTGCAAT CCTGGC)_1 → 2_	Coding (59/753 nt)	*fnr*	DNA-binding transcriptional dual regulator FNR
MC JC	1,978,503	Δ776 bp		*insB-5*–*insA-5*	*insB-5, insA-5*
RA	2,173,363	Δ2 bp	Pseudogene (915-916/1358 nt)	*gatC*	Galactitol-specific PTS enzyme IIC component
RA	3,560,455	+G	Pseudogene (151/758 nt)	*glpR*	DNA-binding transcriptional repressor GlpR
RA	4,296,381	+GC	Intergenic (+587/+55)	*gltP* → /←*yjcO*	Glutamate/aspartateH(+) symporter GltP/Sel1 repeat-containing protein YjcO
Mutations identified in LFB34					
JC	924,581	+AAGA	Coding (1,318/2,277 nt)	*clpA*	ATP-dependent Clp protease ATP-binding subunit ClpA
RA	4,636,768	A → G	I25V (ATT → GTT)	*creC*	Sensory histidine kinase CreC
RA	880,655	C → T	Intergenic (-175/-72)	*gstB* ←/ → *dacC*	Glutathione S-transferase GstB/D-alanyl-D-alanine carboxypeptidase DacC
RA	4,198,232	G → T	A173S (GCC → TCC)	*hemE*	Uroporphyrinogen decarboxylase
JC JC	2,375,828	IS*186* (–) +4 bp	Coding (132-135/963 nt)	*menC*	O-succinylbenzoate synthase
RA	2,535,358	A → T	E431D (GAA → GAT)	*ptsI*	PTS enzyme I
JC JC	2,313,540	Δ2 bp:: IS*186* (+) +6 bp	Coding (53-58/2673 nt)	*rcsD*	RcsD phosphotransferase
RA	3,184,498	C → A	intergenic (-32/-342)	*ribB* ←/ → *ubiK*	3,4-dihydroxy-2-butanone-4-phosphate synthase/ubiquinone biosynthesis accessory factor UbiK
JC JC	3,184,694	Δ1 bp:: IS*186* (–) +6 bp	Intergenic (-228/-141)	*ribB* ←/ → *ubiK*	3,4-dihydroxy-2-butanone-4-phosphate synthase/ubiquinone biosynthesis accessory factor UbiK
RA	3,214,815	T → A	I590N (ATC → AAC)	*rpoD*	RNA polymerase, sigma 70 (sigma D) factor
MC JC	3,823,752	Δ47 bp	Coding (1,353-1,399/2109 nt)	*spoT*	Bifunctional (p)ppGpp synthase/hydrolase SpoT
RA	1,370,981	C → T	R256W (CGG → TGG)	*ycjM*	Glucosylglycerate phosphorylase
JC JC	1,808,767	IS*1* (+) +9 bp	Coding (459-467/537 nt)	*yniB*	Uncharacterized protein YniB
Mutations identified in LFC35					
JC JC	2,536,312	Δ1 bp:: IS*186* (+) +6 bp:: Δ1 bp	Coding (479-484/510 nt)	*crr*	Enzyme IIA(Glc)
RA	663,741	T → C	T75A (ACC → GCC)	*dacA*	D-alanyl-D-alanine carboxypeptidase DacA
RA	439,441	A → G	L246P (CTG → CCG)	*dxs*	1-deoxy-D-xylulose-5-phosphate synthase
RA	4,300,964	C → A	G23C (GGC → TGC)	*mdtP*	Putative multidrug efflux pump outer membrane channel
JC JC	2,375,828	IS*186* (–) +4 bp	Coding (132-135/963 nt)	*menC*	O-succinylbenzoate synthase
RA	665,953	G → A	P121S (CCA → TCA)	*mrdB*	SEDS family protein MrdB
RA	3,163,576	T → A	Intergenic (-95/+139)	*plsC* ←/←*parC*	1-acylglycerol-3-phosphate O-acyltransferase/DNA topoisomerase IV subunit A
RA	2,316,357	G → A	G61S (GGC → AGC)	*rcsB*	DNA-binding transcriptional activator RcsB
JC	435,049	(CCAAAGCTGG CA)_1 → 2_	Coding (403/471 nt)	*ribE*	6,7-dimethyl-8-ribityllumazine synthase
RA	3,377,240	T → G	T61P (ACC → CCC)	*sspA*	Stringent starvation protein A
RA	1,674,374	G → T	L154F (TTG → TTT)	*tqsA*	Autoinducer 2 exporter
RA	3,052,602	A → T	V306E (GTG → GAG)	*ubiH*	2-octaprenyl-6-methoxyphenol hydroxylase
MC JC	2,033,316	Δ59 bp	Coding (111-169/366 nt)	*drpB*	Putative inner membrane protein
RA	4,075,676	(G)_5 → 4_	Coding (124/600 nt)	*yihX*	Alpha-D-glucose-1-phosphate phosphatase

**Figure 6:**
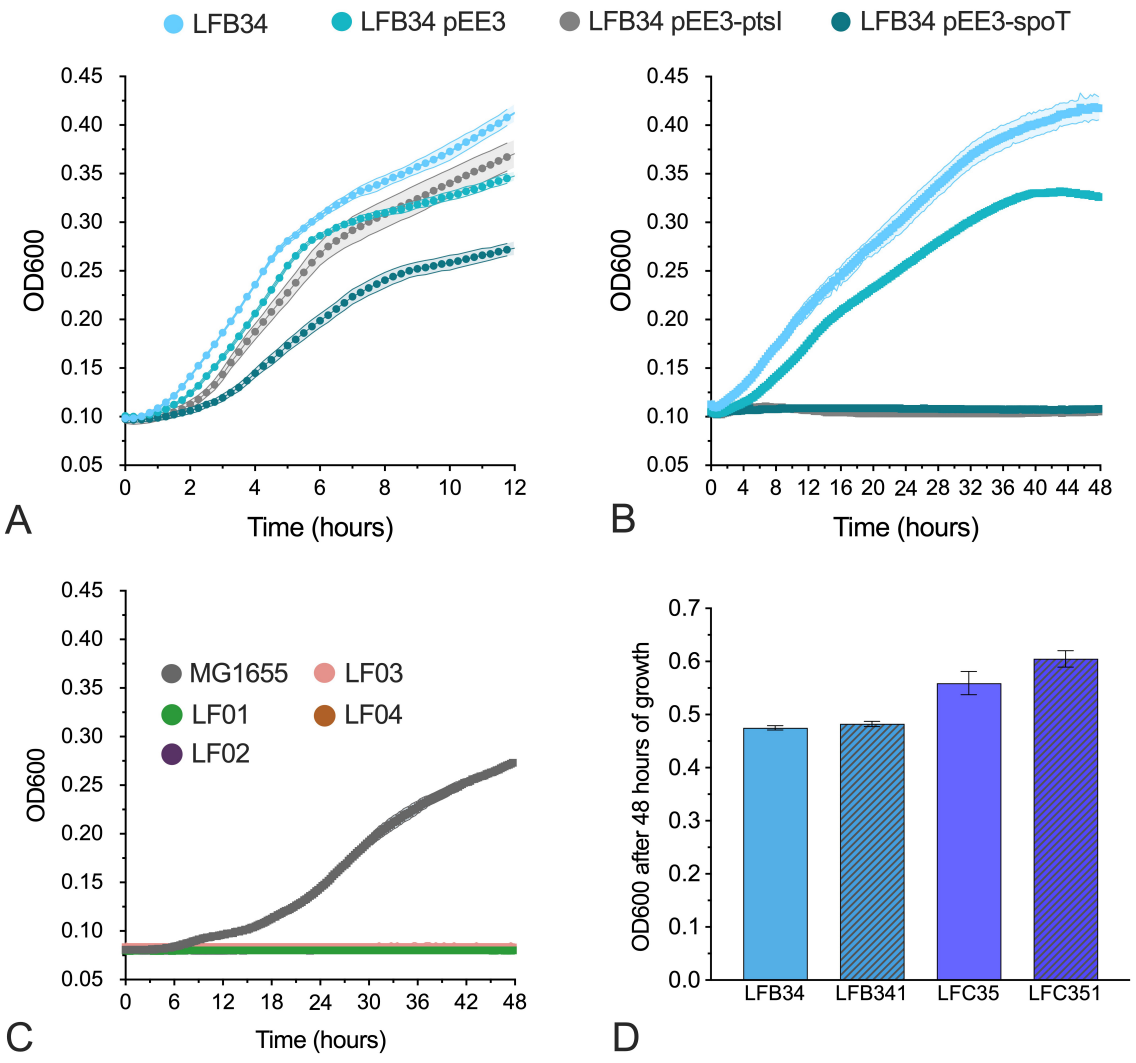
Genetic analyses demonstrated that *ptsI* and *spoT* mutations underpinned MgCl_2_ resistant growth in LFB34, whereas *rcs* genes were essential for tolerance in MG1655. Growth of LFB34 and complemented strains in **(A)** LB and **(B)** 350 mM MgCl_2_ LB. **(C)** Deletion of necessary genes for *rcs* function in the ancestral strain resulted in no growth in 250 mM MgCl_2_ LB. **(D)** Deletion of the same genes in the evolved strains does not impair growth ability in 350 mM MgCl_2_ LB. Points on growth curves represent the mean of three biological replicates and the shaded areas show one standard deviation. Bars represent the mean OD600 of three biological replicates after 48 h of growth and error bars show one standard deviation. LF01 = MG1655 Δ*rcsB*; LF02 = MG1655 Δ*rcsC*; LF03 = MG1655 Δ*rcsD*; LF04 = MG1655 Δ*rcsF*; LFB341 = LFB34, *rcsBCD*::kan; LFC351 = LFC35, *rcsBCD*::kan.

Based on proteomic and comparative genomic analyses, we hypothesized that activation of the Rcs system is no longer adaptive for growth in the evolved strains but remains critical in the ancestor (Supplementary Figure S2; Supplementary Table S2). To test this hypothesis, *rcs* gene deletion and insertion mutants were generated in MG1655, LFB34, and LFC35, and their growth was evaluated in the presence of magnesium chloride. Single deletions of *rcs* genes confirmed that the Rcs system is important for growth under magnesium chloride stress in MG1655, as none of these strains exhibited growth in 250 mM MgCl_2_ LB after 48 h (Figure 6). In contrast, deletion of *rcsBCD* in the evolved strains had no effect on growth in 350 mM MgCl_2_ LB. This surprising result suggests that during laboratory evolution later mutations in both ALE lineages superseded the need for changes in Rcs signaling for MgCl_2_ tolerant growth (Figure 6). The *rcsD* gene was restored in the LFB34 chromosome to generate strain LFB342 which showed no change in magnesium chloride tolerant growth indicating that restoration of Rcs system function does not impact MgCl_2_ resistance in this genetic background. A CRISPR/Cas9 approach was used to generate strain LF05 (MG1655 *rcsB* G61S), which was also able to grow in 250 mM MgCl_2_ LB, indicating that the LFC35 *rcsB* mutation likely does not result in a loss of function (data not shown). Based on these results we conclude that the activation of the Rcs system is important for the growth of the ancestral strain MG1655 at elevated MgCl_2_ concentrations but is not required in the evolved strains. This suggests that the other mutations acquired by the latter supersede the need for Rcs function.

### MgCl_2_ tolerance in LFB34 is associated with altered PTS^*Sugar*^ and stringent response functions

Mutations in genes associated with carbohydrate transport and stress physiology were identified in both evolved strains, including mutations affecting the sugar phosphotransferase (PTS^Sugar^) system in LFB34 and LFC35. In LFB34, a conservative SNP resulting in a glutamic acid to aspartic acid substitution (E431D) was found in *ptsI*, which encodes PtsI (Enzyme I) of the PTS^Sugar^ system (Deutscher et al., 2014). Because this SNP occurs in a magnesium ion binding site required for phosphoenolpyruvate (PEP) interaction (Teplyakov et al., 2006), it could plausibly impair PEP dependent phosphorylation and thereby inhibit the PTS^Sugar^ cascade. In LFC35, a MOB mutation was identified in *crr*, which encodes the PTS^Sugar^ intermediate phosphotransferase protein Crr, likely resulting in loss of function. KEIO strains were evaluated revealing improved growth of JW2409 (BW25113 *ptsI*::kan) and JW2410 (BW25113 *crr*::kan) under magnesium chloride stress, suggesting that mutations in PTS^Sugar^ may be adaptive under MgCl_2_ stress (Supplementary Figure S3). Improved growth was also found in strain LF09 (MG1655 *ptsI*::kan), but unlike the ALE strains, this strain was unable to grow in 350 mM MgCl_2_ LB (data not shown). We also identified a frameshift mutation in the LFB34 *spoT* gene, encoding the bifunctional (p)ppGpp synthase/hydrolase enzyme, likely resulting in a loss of function. Because *SpoT* plays a central role in the stringent response, this allele was further investigated (Bange et al., 2021; Spira and Ospino, 2020; Montero et al., 2014). Complementation of LFB34 with *spoT* cloned into the plasmid vector pEE3 abolished growth in 350 mM MgCl_2_ LB relative to the empty vector control (Figure 6). The empty pEE3 vector reduced final biomass relative to plasmid free LFB34, likely reflecting vector associated burden and/or kanamycin selection, as plasmid maintenance can impose fitness costs on *E. coli* K-12 strains (Dahlberg and Chao, 2003). Accordingly, complementation phenotypes were interpreted relative to the corresponding empty pEE3 vector control. The loss of growth following *spoT* complementation supports that loss or reduction of SpoT-mediated stringent response activity is critical to the growth of LFB34 at high MgCl_2_ concentrations. To assess whether loss of SpoT function is sufficient to confer improved MgCl_2_ tolerance, strain LF08 (MG1655 *spoT*::kan) was constructed and evaluated; however, LF08 retained ancestral strain MgCl_2_ sensitivity (data not shown). Thus, the lack of SpoT function is necessary but insufficient for adaptation in the evolved strain LFB34.

## Discussion

### Envelope modifications in the evolved strains may aid in resistance to chaotropic stress

To investigate how *E. coli* adapts to chaotropic magnesium chloride stress, ALE was performed by serially propagating MG1655 in LB with progressively increasing concentrations of MgCl_2_. Two evolved growth defective strains were obtained which had enhanced growth in MgCl_2_ and various chaotropic salts, but not NaCl, indicating a distinct chaotolerant phenotype. This phenotype underscores that chaotropic stress differs fundamentally from osmotic stress imposed by NaCl. Consistent with this conclusion, mutations commonly observed in *E. coli* ALE experiments utilizing increased NaCl levels (*rpoB, topA, fes, fepA, rph, pykF, proV, mreB*, and *nag*) were absent from both MgCl_2_ ALE strains (Cesar et al., 2020; Dragosits et al., 2013; Lennen et al., 2023; Wang et al., 2018; Winkler et al., 2014; Zorraquino et al., 2016). However, both types of selection can result in cell wall changes. In the MgCl_2_ adapted strains, mutations were identified in or near genes associated with peptidoglycan biosynthesis, peptidoglycan maturation, cell division, and phospholipid biosynthesis, including *dacA, dacC, mrdB, drpB*, and *plsC* (Peters et al., 2016; Spratt and Strominger, 1976; Stoker et al., 1983; Zhang and Rock, 2008; Yahashiri et al., 2020). The significance of the *dacA* mutation is underscored by the inability of strain JW0627 (BW25113 *dacA*::kan) to grow under MgCl_2_ stress (Supplementary Figure S3). In addition, *mrdA* mutations commonly arise in ALE experiments targeting osmotic stress tolerance and may contribute to resistance by altering cell wall structure (Matsuzawa et al., 1989; Winkler et al. 2014). The *dacA* and *mrdB* mutations are also noteworthy because of the role of these genes in the control of cell shape (Meeske et al., 2016; Nelson and Young, 2000; Winkler et al., 2014). The cell morphology data in Figure 3 provide physiological support for this interpretation. In LB, the evolved strains diverged from MG1655 in opposite ways, with LFB34 exhibiting a significantly smaller cell volume and LFC35 exhibiting a substantially larger cell volume. Upon transition to MgCl_2_ amended LB, MG1655 displayed pronounced filamentation, consistent with disruption of normal cell division under stress. In contrast, LFB34 remained comparatively small and rod shaped, while LFC35 shifted from enlarged rods in LB to shorter, narrower, curved cells under MgCl_2_ stress. These divergent morphological responses suggest that MgCl_2_ adaptation did not converge on a single cell size/shape, but instead produced lineage specific changes in morphology. This interpretation is consistent with the central role of peptidoglycan synthesis and remodeling in bacterial morphology and supports the idea that the evolved strains altered envelope architecture and division control through distinct routes (Egan et al., 2020). The intergenic *plsC* / *parC* mutation may alter expression of *plsC* (1-acylglycerol-3-phosphate O-acyltransferase) which is critical for phospholipid biosynthesis (Zhang and Rock, 2008). PlsC abundance was increased in LFC35 relative to MG1655 in both LB and LB250, with log2 fold changes of 2.41 and 1.44, respectively (Supplementary Table S2). Because PlsC is required for phosphatidic acid formation, this increase may reflect membrane remodeling under MgCl_2_ stress, a process previously implicated in osmotic tolerance (Romantsov et al., 2009; Rowlett et al., 2017). Additional evidence for envelope modification in LFC35 is that it is resistant to P1 phage infection, consistent with outer membrane modification (Figure 5; Supplementary Figure S4; Meyer et al., 2012). These membrane changes may be complemented by increased polyamine biosynthesis, as CadA, SpeA, SpeB, and SpeC, were upregulated in both evolved strains. Polyamines contribute to tolerance against diverse stressors (Akhova et al., 2021; Das and Misra, 2004; Rhee et al., 2007) but have also been shown to reduce the permeability of cation-selective porins (Samartzidou and Delcour, 1999; Samartzidou et al., 2003). Increased polyamine biosynthesis could therefore support MgCl_2_ tolerance by modifying outer membrane permeability and limiting excessive cation influx under high MgCl_2_ conditions. Although the potential fitness advantage of altered cell morphogenesis under MgCl_2_ stress remains unresolved, the combined imaging, genomic, and proteomic data collectively suggest that envelope modification is critical for resistance to chaotropic stress, which is known to substantially impact membrane integrity (Cray et al., 2013; Hallsworth et al., 2007).

### Divergent impact of the Rcs system in MgCl_2_ resistance after ALE

Genomic, proteomic, and genetic analyses indicated that the Rcs system is important for MgCl_2_ resistance in MG1655 but dispensable in both evolved strains (Figure 6; Supplementary Figure S2; Supplementary Table S2). This finding is intriguing given the well-known role of the Rcs system in response to hyperosmotic stress (Majdalani and Gottesman, 2005; Sledjeski and Gottesman, 1996; Takeda et al., 2001; Wall et al., 2018). Proteomics analysis revealed clear and strong activation of Rcs genes only in MG1655 (Supplementary Table S2; Supplementary Figure S2). The presence of *rcs* mutations in both evolved strains is notable, as such changes rarely arise during ALE to osmotic stress (Lennen et al., 2023; Winkler et al., 2014). It is also important to note that LFC35 harbors a loss of function mutation in *drpB*, which encodes a non-essential cell division protein recently shown to activate Rcs signaling independently of RcsF (Petchiappan et al., 2024). Because DrpB has also been reported to be dispensable during cell division under osmotic stress (Yahashiri et al., 2020), the improved MgCl_2_ growth observed upon *drpB* deletion (Supplementary Figure S3) suggests that loss of DrpB may improve fitness under chaotropic stress by reducing interference with cell division machinery and limiting maladaptive Rcs activation in the evolved background. Collectively, our findings indicate that the evolved strains no longer rely on Rcs signaling for MgCl_2_ resistance. One possibility is that the Rcs response in the ancestor mitigates MgCl_2_ stress only up to an intermediate threshold, whereas growth at the higher MgCl_2_ concentrations selected during ALE required additional adaptations that bypassed the need for this pathway. The dispensability of the Rcs system in this context underscores the power of adaptive laboratory evolution to reveal unanticipated routes to stress tolerance.

### Potential roles of the stringent response and PTS^*Sugar*^ deficiency in MgCl_2_ stress resistance

Our data strongly indicates that mutations in *spoT* and *ptsI* play a significant role in adaptation to chaotropic stress in LFB34 (Figure 6). SpoT plays a key role in modulating the stringent response, cell size, and overall metabolic state by adjusting the intracellular concentrations of the alarmone (p)ppGpp, guanosine 3′-diphosphate 5′-di/triphosphate (Büke et al., 2022; Fernández-Coll and Cashel, 2020; Hauryliuk et al., 2015; Patacq et al., 2020; Potrykus and Cashel, 2008). Accumulation of the alarmone depresses growth and increases stress tolerance by redirecting transcriptional and translational programs from biosynthesis and rapid proliferation toward stress protection and resource conservation (Bryson et al., 2020; Horvatek et al., 2020; Kim et al., 2018; Pacios et al., 2020; Qi et al., 2024). RelA (GDP/GTP pyrophosphokinase) produces the majority of (p)ppGpp in the cell but lacks hydrolase activity, while SpoT uniquely has dual functionality, with strong hydrolase and weak synthetase activity (Bange et al., 2021; Fernández-Coll and Cashel, 2020; Spira and Ospino, 2020). The Δ47 bp deletion in the coding region of the LFB34 *spoT* allele would likely eliminate SpoT function, consistent with (p)ppGpp accumulation contributing to the observed decrease in growth rate and increased MgCl_2_ tolerance. Proteomics data further supports a stringent response in LFB34 as levels of the hibernation factors Rmf, Hpf, and RaiA were dramatically higher relative to MG1655 (Supplementary Table S2). The expression of the genes encoding these proteins is strongly induced by (p)ppGpp and have documented roles in universal bacterial stress tolerance by curbing translation rates to liberate energy for survival rather than growth (el-Sharoud and Niven, 2005; Garay-Arroyo et al., 2000; Prossliner et al., 2021) which could contribute to chaotropic stress resistance. Importantly, complementation of LFB34 with wild-type *spoT* abolished growth in 350 mM MgCl_2_ LB, strongly supporting that loss of SpoT function and consequent dysregulation of (p)ppGpp homeostasis are central to LFB34 growth at high MgCl_2_ concentrations, potentially through (p)ppGpp accumulation due to lack of SpoT hydrolase activity (Figure 6).

Our results also provide further support to previous models implicating *ptsI* and the PTS^Sugar^ system in bacterial stress resistance. *E. coli ptsI* mutants have been shown to exhibit pan-stress tolerance linked to reduced cAMP levels and altered central carbon metabolism, resulting in decreased respiration, reduced ROS-associated macromolecular damage, and increased survival (Kohanski et al., 2007; Zeng et al., 2022). In LFB34, the loss of MgCl_2_ tolerance following complementation with wild-type *ptsI* supports a similar model in which the mutant *ptsI* allele contributes directly to stress resistance, potentially by abolishing PTS^Sugar^ activity and consequent ROS accumulation (Figure 6). The LFC35 *crr* mutation, which likely results in loss of gene function, further implicates the PTS^Sugar^ system in MgCl_2_ tolerance, as *E. coli* Δ*crr* strains exhibit pan-stress tolerance through the same mechanism as *ptsI* mutants (Zeng et al., 2022). Consistent with this model, ROS measurements performed using the approach of Zeng et al. (2022) indicated greater ROS accumulation in the ancestral strain than in the evolved strains following H_2_O_2_ exposure, suggesting that altered PTS^Sugar^ activity may be adaptive under chaotropic stress by reducing ROS accumulation, a phenotype particularly relevant given that chaotropic stress has been linked to oxidative damage (Supplementary Figure S5; Cray et al., 2015; Hamill et al., 2020). Importantly, neither loss of *spoT* nor mutation of *ptsI* alone was sufficient to confer MgCl_2_ tolerance in the ancestral background, suggesting that these alleles contribute to resistance specifically within the evolved genetic context of LFB34. Thus, MgCl_2_ tolerance likely reflects a complex polygenic phenotype requiring coordinated changes in the stringent response, attenuation of Rcs activation, envelope remodeling, and mitigation of oxidative damage.

Consistent with the latter point, proteomic enrichment of metal binding and iron sequestration proteins in the evolved strains further supports a role for limiting ROS associated damage. In particular, Bfr and FtnA, major *E. coli* iron-storage proteins involved in iron sequestration and oxidative stress tolerance (Abdul-Tehrani et al., 1999; Bitoun et al., 2008), were significantly enriched in the evolved strains (Supplementary Table S2). Elevated levels of these iron-storage complexes may reduce intracellular labile iron, including reactive ferrous iron, thereby limiting Fenton chemistry, one of the major sources of ROS associated macromolecular damage (Abdul-Tehrani et al., 1999; Andrews, 1998; Bitoun et al., 2008; Kim et al., 2018; Kohanski et al., 2007). Several copper and zinc binding proteins, including ZraP, RclA, CutA, and CueO, which are implicated in metal ion detoxification, antibiotic resistance, and oxidative stress tolerance (Supplementary Table S2; Baek et al., 2020; Derke et al., 2020; Fong et al., 1995; Grass and Rensing, 2001; Rome et al., 2018) were also upregulated in the evolved strains. Together, these observations suggest that remodeling central carbon metabolism and mitigating oxidative damage are key strategies for adaptation to chaotropic stress.

## Data Availability

The datasets presented in this study can be found in online repositories. The names of the repository/repositories and accession number(s) can be found in the article/Supplementary material.
