# Supplementary material for: Adaptive laboratory evolution of E. coli to magnesium chloride stress reveals noncanonical routes to broad chaotropic salt resistance
Source: Front Microbiol. 2026 Jul 23;17:1898632. doi: 10.3389/fmicb.2026.1898632 (PMC13446271; doi:10.3389/fmicb.2026.1898632)
Supplement: Supplementary file 1 [file Supplementary_file_1.docx]

**Supplementary Methods**

**Mass spectrometry acquisition for the construction of the DIA library**

Each sample (0.2 µg) was analyzed by ultra-high pressure liquid chromatography (UPLC) coupled with tandem mass spectroscopy (LC-MS/MS) using nano-spray ionization. The nanospray ionization experiments were performed using a timsTOF Pro 2 hybrid mass spectrometer (Bruker, San Jose, CA, USA) interfaced with nano-scale reversed-phase UPLC (Evosep One, Odense, Denmark). The Evosep method ‘30 samples per day’ was utilized using a 15 cm x 150 µm reverse-phase column packed with 1.5 µm C18-beads (Bruker, San Jose, CA, USA) at 58˚C. The analytical columns were connected with a fused silica ID emitter (10 µm ID; Bruker Daltonics, San Jose, Cam USA) inside a nanoelectrospray ion source (Bruker, San Jose, CA, USA). The mobile phases consisted of 0.1% formic acid (FA) as solution A and 0.1% FA, 99.9% acetonitrile as solution B. The mass spectrometry setting ‘PASEF method for standard proteomics’ for the timsTOF Pro 2 was used. The values for mobility-dependent collision energy ramping were set to 95 eV at an inversed reduced mobility (1/k0) of 1.6 V s/cm^2^ and 23 eV at 0.73 V s/cm^2^. Collision energies were linearly interpolated between these two 1/k0 values and kept constant above or below. No merging of TIMS scans was performed. Target intensity per individual PASEF precursor was set to 20,000. The scan range was set between 0.6 and 1.6 V s/cm^2^ with a ramp time of 166 ms. 14 PASEF MS/MS scans were triggered per cycle (2.57 s) with a maximum of seven precursors per mobilogram. Precursor ions in an m/z range between 100 and 1700 with charge states ≥3+ and ≤8+ were selected for fragmentation. Active exclusion was enabled for 0.4 min (mass width 0.015 Th, 1/k0 width 0.015 V s/cm^2^). Peptide identifications were carried out using Peaks Studio 11 version 12.5 (Bioinformatics Solutions, Ontario, Canada) against the *Escherichia coli* K-12 MG1655 (GenBank no. U00096.3) proteome, and peptides with a false discovery rate (FDR) cutoff of 1% were selected for the DIA (data-independent acquisition) library file.

**Microscopy and cell size analysis:**

Emitted light was first passed through a 735 nm low-pass dichroic filter (Chroma Technology Corporation, Rockingham, VT, USA) then through a 530/43 nm bandpass filter before reaching the avalanche photodiode detector (Perkin Elmer, Waltham, MA, USA). All fluorescence intensity imaging was done via raster scanning with a 0.04 ms pixel dwell time in the 256 x 256 pixel FOV. NADH was used to determine cell size because it is homogenously distributed throughout cells and is an intrinsic fluorophore, so no genetic manipulation in the cells was required.

All cell size images were analyzed using a custom matlab script. The matlab script allows for fluorescence intensity thresholding of each FOV, allowing the user to best visualize all cells before segmentation. Next, an AI based on U-net deep learning architecture and resnet-50 was used to segment individual cells after denoising with BM3D. To improve segmentation, the model was initially trained using manually segmented MG1655 cells. Then, weight maps were introduced to encourage learning separation between densely packed FOVs. Finally, the model allows the user to customize the segmentation by deleting poorly masked cells, adding masks that were missed by the AI, and splitting cells that were improperly segmented as one cell. When new cells are added, the user can specify the length and width of the rod shaped mask. After segmentation, cell volume was determined by assuming each cell is composed of a cylindrical prism capped by a hemisphere on each side (Reshes et al., 2008). Thus, the 2D stadium shape of each *E. coli* can be extrapolated to 3D space. Using the length (L) and the width (W), the volume (V) was calculated as indicated below. Importantly, cells must be rod shaped and straight for the volume calculation to be accurate. However, the model can segment cells with variable lengths and widths.

$$V=\pi\left( \frac{W}{2} \right)^{2}\left( L-W \right)+\frac{4}{3}\pi{(\frac{W}{2})}^{3}$$

**Supplementary Figures**

Supplementary Figure 1: Growth of the evolved strains is severely impaired in minimal M9 medium. Colored bars show OD600 after 20 hours of growth and patterned, outlined, bars indicate OD600 after 40 hours of growth. Bars represent the average OD600 of three biological replicates and error bars show one standard deviation.

Supplementary Figure 2: Heat map of select DEPs sorted by GO function implicated in the osmotic stress response, cell division, capsule/lipopolysaccharide biosynthesis and transport, and colanic acid biosynthesis in the LB250 condition. Columns represent the strain, rows indicate protein, and GO categories are color coded on the left. Warmer colors indicate increased protein abundance and cooler colors indicate decreased protein abundance when compared across all three strains indicated by Z-score. Log2 fold change values can be found in Table S2 and Supplementary File 1.

Supplementary Figure 3: Growth curves of BW25113 and select Keio strains in 250 mM MgCl_2_ LB. Points show the mean of three biological replicates and shaded areas indicate one standard deviation. JW2410=BW25113 *crr*::kan; JW0627=BW25113 *dacA*::kan; JW2409 (BW25113 *ptsI*::kan).

Supplementary Figure 4: LFC35 is completely resistant to P1 phage. Top agar plates of the ancestral and evolved strains (indicated above) with (+P1) and without (Control) the addition of P1 phage. Opaque plates indicate confluent growth. A blank LB plate is shown on the right as a comparison.

Supplementary Figure 5: Fluorescence intensity overtime of MG1655, LFB34, and LFC35 cells stained with Carboxy-H2DCFDA upon exposure to 2 mM H_2_O_2_. Points represent the mean of three biological replicates, and the shaded areas indicates the standard deviation. Unstained and stained controls showed little fluorescence.

**Supplementary Tables:**

| **Primer name** | **Sequence (5’ → 3’)** |
| --- | --- |
| rcsB_KO_F | CATTGACAGTTATGTCAAGAGCTTGCTGTAGCAAGGTAGCCTATTACATGGTGTAGGCTGGAGCTGCTTC |
| rcsB_KO_R | CTAACGCGTCTTATCTGGCCTACAGGTGATTAGTCTTTATCTGCCGGACTATGGGAATTAGCCATGGTCC |
| rcsB_Ck_F | GCTCATCGTCTTAAAGGCGT |
| rcsB_Ck_R | CGTTGGCTGAAGAGAAGCA |
| rcsC_KO_F | CGCTACCTTAACCACACTCCATCGGTCACCTGAGGCGGAGCTTCGCCCCTGTGTAGGCTGGAGCTGCTTC |
| rcsC_KO_R | ATCTGGCATTTGCACTGAATGCCGGATGCGGCGTAAACGCCTTATCCGTCATGGGAATTAGCCATGGTCC |
| rcsC_Ck_R | CCAGATATAGCCGAGGATTT |
| rcsD_KO_F | TATACTGCCCTATCACTTCGCGAAGTTTTAACAGGTCATAAACACGAATGGTGTAGGCTGGAGCTGCTTC |
| rcsD_KO_R | TCATATTGTTCATGTAATAGGCTACCTTGCTACAGCAAGCTCTTGACATAATGGGAATTAGCCATGGTCC |
| rcsD_Ck_F | GAGTCTGGAATTTCACACTG |
| rcsD_Ck_R | ACAAGACTATCGGATGGTCA |
| rcsF_KO_F | CAAGCTCCTGATTCAATATTGACGTTTTGATCATACATTGAGGAAATACTGTGTAGGCTGGAGCTGCTTC |
| rcsF_KO_R | TTATAGGGCGAGCGAATAACGCCTATTTGCTCGAACTGGAAACTGCTCATATGGGAATTAGCCATGGTCC |
| rcsF_Ck_F | TGCCGAGCTTCAGAATCAC |
| rcsF_Ck_R | TATGCCAGCCAGATTGGC |
| ptsI_KO_F | GAGTAATTTCCCGGGTTCTTTTAAAAATCAGTCACAAGTAAGGTAGGGTTGTGTAGGCTGGAGCTGCTTC |
| ptsI_KO_R | ACAAACCCATGATCTTCTCCTAAGCAGTAAATTGGGCCGCATCTCGTGGAATGGGAATTAGCCATGGTCC |
| ptsI_Ck_F | GTTACCATTACCGCTCCGAA |
| ptsI_Ck_R | CGAAGTGGACGAACAGTTCAA |
| rcsB_N20_S | AGCGATTACCGATCTCTCCATGCC |
| rcsB_N20_AS | AAACGGCATGGAGAGATCGGTAAT |
| rcsB_donor_F | CCAGGTCTCAGTGCGTAGCAAGGTAGCCTATTAC |
| rcsB_donor_F | CCAGGTCTCAGAGCCTTATCTGGCCTACAGGTGA |
| Part_A_fwd_kan | CAGTGACAACGTCGAGCAC |
| Part_A_rev_kan | TTACTCGAGTTTGGATGCTAAGCA |
| pRED_frag2_fwd | GGATCTCCAGGCATCAAATAAAAC |
| Part_2_rev_kan | AAGCGGGAAGGGACTGG |
| pRED_Dummy_4919-4938_F | AAGATCGGTCAGGGTAAAGC |
| pRED_Dummy_5398-5417_R | AGAGCGTTCACCGACAAACA |
| pRED_Dummy_10649-10668_F | GGCGCAATCACTTTCGTCTA |
| pRED_Dummy_11176-11195_R | GGTGCTTATGTGATCTACGT |
| Gent_Ck_F | ATTACGGTGACGATCCCGC |
| Gent_Ck_R | ATGTTGGGAGTAGGTGGCTACG |
| rcsD_60bp_up_F | ACACTGTACCCTTTATACTG |
| rcsD+60bp down_R | ACTATCGGATGGTCATCG |
| pEE3-fwd | GTTAATTGCGCGCTTGGC |
| pEE3-rev | GTACTGCGATGAGTGGCA |
| pEE3_spoT_fwd | TACGCCCCGCCCTGCCACTCATCGCAGTACACCTGATTTCAGTATCATG |
| pEE3_spoT_rev | GACCATGATTACGCCAAGCGCGCAATTAACTTAATTTCGGTTTCGGGTG |
| ptsI_pEE3_fwd | CAACAGTAATGCCAGCTTGT |
| ptsI_pEE3_rev | GCATCTCGTGGATTAGCAGA |

Supplementary Table 1: Primers used in this study.

| Uniprot ID | Protein | Description | 1 | 2 | 3 | 4 |
| --- | --- | --- | --- | --- | --- | --- |
| **Proteins involved in colanic acid (CA) biosynthesis** | | | | | | |
| P24174 | CpsB | Mannose-1-phosphate guanylyltransferase |  |  | -4.57 | -4.27 |
| P32055 | Fcl | GDP-L-fucose synthase |  |  | -4.01 | -6.11 |
| P09147 | GalE | UDP-glucose 4-epimerase |  |  | -3.44 | -1.49 |
| P0AC88 | Gmd | GDP-mannose 4,6-dehydratase |  |  | -7.94 | -6.64 |
| P32056 | Gmm | GDP-mannose mannosyl hydrolase |  |  | -4.65 | -4.62 |
| P00946 | ManA | Mannose-6-phosphate isomerase |  |  | -2.52 | -2.95 |
| P76373 | Ugd | UDP-glucose 6-dehydrogenase |  |  | -8.11 | -7.04 |
| P77414 | WcaA | Putative colanic acid biosynthesis glycosyl transferase |  |  | -4.60 | -8.41 |
| P71237 | WcaC | Putative colanic acid biosynthesis glycosyl transferase |  |  | -5.53 | -5.81 |
| P71239 | WcaE | Putative colanic acid biosynthesis glycosyl transferase |  |  | -4.89 | -3.65 |
| P32057 | WcaI | Putative colanic acid biosynthesis glycosyl transferase |  |  | -3.08 | -3.44 |
| P71241 | WcaJ | UDP-glucose:undecaprenyl-phosphate glucose-1-phosphate transferase |  |  | -4.86 | 0.49 |
| P71242 | WcaK | Colanic acid biosynthesis protein |  |  | -4.73 | -4.01 |
| P71243 | WcaL | Putative colanic acid biosynthesis glycosyltransferase |  |  | -4.62 | -5.27 |
| P71244 | WcaM | Colanic acid biosynthesis protein |  |  | -4.34 | -1.65 |
| P0A930 | Wza | Putative polysaccharide export protein |  |  | -5.09 | -6.28 |
| P0AAB2 | Wzb | Low molecular weight protein-tyrosine-phosphatase |  |  | -4.77 | -1.50 |
| P76387 | Wzc | Tyrosine-protein kinase |  |  | -5.38 | -4.15 |
| **ATP synthase subunits** | | | | | | |
| P0ABB0 | AtpA | ATP synthase subunit alpha | -1.91 | -0.65 | -2.65 | -1.97 |
| P0A6E6 | AtpC | ATP synthase epsilon chain |  |  | -2.27 | -0.37 |
| P0ABB4 | AtpD | ATP synthase subunit beta | -1.92 | -0.53 | -2.58 | -1.85 |
| P68699 | AtpE | ATP synthase subunit c | -4.10 | -1.88 | -7.11 | -5.88 |
| P0ABA0 | AtpF | ATP synthase subunit b | -1.91 | -0.87 | -2.88 | -2.46 |
| P0ABA6 | AtpG | ATP synthase gamma chain | -1.60 | -0.58 | -2.79 | -1.21 |
| P0ABA4 | AtpH | ATP synthase subunit delta |  |  | -1.57 | -0.03 |
| **Proteins involved in phospholipid biosynthesis, and peptidoglycan biosynthesis, maturation, and recycling** | | | | | | |
| P36548 | AmiA | N-acetylmuramoyl-L-alanine amidase | 0.33 | 1.90 | 1.84 | 3.13 |
| P26365 | AmiB | N-acetylmuramoyl-L-alanine amidase |  |  | 3.00 | 2.05 |
| P63883 | AmiC | N-acetylmuramoyl-L-alanine amidase |  |  | 1.84 | 1.50 |
| P75820 | AmiD | N-acetylmuramoyl-L-alanine amidase |  |  | -0.15 | -1.19 |
| P0A6L2 | DapA | 4-hydroxy-tetrahydrodipicolinate synthase | 0.23 | 1.80 | 1.57 | 2.73 |
| P04036 | DapB | 4-hydroxy-tetrahydrodipicolinate reductase |  |  | 1.59 | 1.69 |
| P0A9D8 | DapD | 2,3,4,5-tetrahydropyridine-2,6-dicarboxylate N-succinyltransferase | 1.23 | 0.47 | 1.94 | 1.75 |
| P0AED7 | DapE | Succinyl-diaminopimelate desuccinylase | -0.32 | 1.20 | 1.37 | 2.54 |
| P39176 | LdtA | L,D-transpeptidase | 2.50 | 1.63 | 3.74 | 1.61 |
| P76193 | LdtE | L,D-transpeptidase | 2.55 | 1.53 | 3.99 | 2.64 |
| P08660 | LysC | Lysine-sensitive aspartokinase 3 | 0.86 | 1.32 | 2.57 | 2.75 |
| P02918 | MrcA | Penicillin-binding protein 1A | -1.37 | 0.00 | -2.24 | -1.53 |
| P02919 | MrcB | Penicillin-binding protein 1B |  |  | -3.65 | -3.74 |
| P0AD65 | MrdA | Peptidoglycan D,D-transpeptidase | -1.16 | -1.20 | -2.58 | -2.52 |
| P0A9X4 | MreB | Cell shape-determining protein | 1.24 | 0.23 | -0.25 | -1.38 |
| P16926 | MreC | Cell shape-determining protein |  |  | -1.06 | -1.87 |
| P0AF18 | NagA | N-acetylglucosamine-6-phosphate deacetylase |  |  | 2.08 | 2.36 |
| P0A759 | NagB | Glucosamine-6-phosphate deaminase |  |  | 2.07 | 2.34 |
| P0AF20 | NagC | N-acetylglucosamine repressor |  |  | 1.70 | 0.82 |
| P09323 | NagE | PTS system N-acetylglucosamine-specific EIICBA component | -1.09 | -2.40 | 1.36 | 0.08 |
| P75959 | NagK | N-acetyl-D-glucosamine kinase |  |  | 3.12 | 4.62 |
| P26647 | PlsC | 1-acylglycerol-3-phosphate O-acyltransferase | -0.48 | 2.41 | -0.90 | 1.44 |
| P23721 | SerC | Phosphoserine aminotransferase | 1.95 | 1.12 | 3.33 | 2.10 |
| P0AGC3 | Slt | Soluble lytic murein transglycosylase | 1.47 | 1.48 | 3.78 | 2.80 |
| **Metal ion binding and membrane protein biogenesis and repair** | | | | | | |
| P66948 | BepA | Beta-barrel assembly-enhancing protease | 0.37 | 3.27 | 2.05 | 3.96 |
| P0ABD3 | Bfr | Bacterioferritin |  |  | 5.17 | 4.08 |
| P36649 | CueO | Multicopper oxidase |  |  | 3.53 | 2.98 |
| P69488 | CutA | Divalent-cation tolerance protein | 2.66 | 2.93 | 3.41 | 2.79 |
| P77202 | DsbG | Thiol:disulfide interchange protein |  |  | 2.85 | 1.78 |
| P0A998 | FtnA | Bacterial non-heme ferritin | -2.60 | -2.19 | 4.05 | 4.00 |
| P76342 | MsrP | Protein-methionine-sulfoxide reductase catalytic subunit | 3.21 | 2.52 | 3.83 | 3.06 |
| P23843 | OppA | Periplasmic oligopeptide-binding protein | 1.22 | 0.68 | 4.48 | 3.25 |
| P0AFL3 | PpiA | Peptidyl-prolyl cis-trans isomerase A | 0.32 | 1.16 | 2.45 | 3.25 |
| P77212 | RclA | Probable pyridine nucleotide-disulfide oxidoreductase | 2.33 | 1.58 | 3.12 | 3.30 |
| P0AEU7 | Skp | Chaperone protein Skp |  |  | 4.88 | 4.38 |
| P0ABZ6 | SurA | Chaperone SurA |  |  | 1.29 | 1.37 |
| P0AAA9 | ZraP | Signaling pathway modulator |  |  | 3.57 | 5.69 |
| **Hibernation factors (HFs) and proteins involved in polyamine biosynthesis** | | | | | | |
| P0A9H3 | CadA | Inducible lysine decarboxylase (LDCI) | 3.22 | 1.54 | 4.69 | 4.41 |
| P0AFX0 | Hpf | Ribosome hibernation promoting factor (HPF) | 1.07 | 1.51 | 3.13 | 3.14 |
| P0AD49 | RaiA | Ribosome-associated inhibitor A | 1.02 | 0.73 | 1.33 | 1.02 |
| P0AFW2 | Rmf | Ribosome modulation factor (RMF) | 5.99 | 7.63 | 4.00 | 4.48 |
| P21170 | SpeA | Biosynthetic arginine decarboxylase (ADC) |  |  | 0.25 | 1.37 |
| P60651 | SpeB | Agmatinase |  |  | 2.00 | 1.98 |
| P21169 | SpeC | Constitutive ornithine decarboxylase | 0.99 | 1.48 | 1.06 | 1.27 |
| P0A7F6 | SpeD | S-adenosylmethionine decarboxylase proenzyme | -0.55 | -1.13 |  |  |

Supplementary Table 2: Select DEPs organized by molecular or biological function identified using EcoCyc (Keseler et al., 2011). Values show log2 fold changes for the following comparisons. (1) LFB34 vs. MG1655 (LB); (2) LFC35 vs. MG1655 (LB); (3) LFB34 vs MG1655 (LB250); and (4) LFC35 vs. MG1655 (LB250). For the full list of DEPS, see Supplementary File 1.

**References**

Keseler, I.M., Collado-Vides, J., Santos-Zavaleta, A., Peralta-Gil, M., Gama-Castro, S., Muñiz-Rascado, L., Bonavides-Martinez, C., Paley, S., Krummenacker, M., Altman, T., Kaipa, P., Spaulding, A., Pacheco, J., Latendresse, M., Fulcher, C., Sarker, M., Shearer, A.G., Mackie, A., Paulsen, I., and Karp, P.D. (2011) EcoCyc: a comprehensive database of *Escherichia coli* biology. *Nucleic Acids research* 39: D583–590. doi: 10.1128/genomeA.00975-14.

Reshes G., Vanounou S., Fishov I., and Feingold M. (2008) Timing the start of division in *E. coli*: a single-cell study. *Physical Biology* 5. doi: 10.1088/1478-3975/5/4/046001.
